# Biportal endoscopic posterior cervical foraminotomy with discectomy for unilateral radicular arm pain due to cervical herniated disc

**DOI:** 10.3171/2024.1.FOCVID23225

**Published:** 2024-04-01

**Authors:** Dong Hwa Heo

**Affiliations:** Department of Neurosurgery, Endoscopic Spine Surgery Center, Chungdam Harrison Spinartus Hospital, Seoul, South Korea

**Keywords:** cervical, radiculopathy, disc disease, foraminotomy, endoscopy, biportal

## Abstract

Recently, biportal endoscopic cervical approaches have been used to treat cervical degenerative disease. Biportal endoscopic posterior cervical foraminotomy with or without discectomy has the advantage of reducing damage to the normal tissues during surgery and enhancing fast recovery after surgery. The biportal endoscopic cervical approach was performed using two portals. The first portal was an endoscopic viewing portal for the spinal endoscope, and the other portal was a working portal for using surgical instruments. The author illustrates the surgical technique of biportal endoscopic posterior cervical foraminotomy with discectomy.

The video can be found here: https://stream.cadmore.media/r10.3171/2024.1.FOCVID23225

## Transcript

### 0:25 Case Presentation.

I am presenting the case of a 40-year-old male patient who is experiencing severe radiating pain in his right arm. Despite undergoing two cervical epidural blocks, there has been no improvement in his symptoms. This intense pain in the right arm has significantly impacted his ability to sleep. Upon neurological examination, it was observed that the radicular pain in the right arm was localized within the C7 dermatome, and the Spurling sign elicited a positive response.

### 0:57 Preoperative MRI.

The preoperative T2 axial and sagittal images revealed a ruptured disc in the right C6–7 region. Particles from the ruptured disc were exerting pressure on the right C7 nerve root, specifically situated in the right paracentral to foraminal area of C6–7.

### 1:17 Patient Position.

During the operation, the patient was positioned prone with a slight flexion in the neck. To ensure protection, a polyurethane foam pad was used to shield the patient’s face and eyes.

### 1:23 Making Two Portals.

Biportal endoscopic posterior cervical foraminotomy necessitates the creation of two distinct portals: one serves as the endoscopic viewing portal, while the other functions as the working portal.^1–4^ The surgical methodology closely resembles minimally invasive microsurgery in most aspects.^2,4^

### 1:54 Intraoperative Fluoroscopic C-Arm Images.

Under C-arm fluoroscopic guidance, two portals are established at the C6 and C7 pedicle levels. One portal is exclusively designated for spinal endoscopy, while the other is specifically allocated for surgical instrument use. To ensure efficient drainage of saline irrigation and facilitate the seamless insertion of surgical instruments, a working sheath is carefully placed within the dedicated working portal.

### 2:23 Overview of Biportal Endoscopic Approach.

The images presented depict an overview of the biportal endoscopic cervical approach. The biportal endoscopic devices bear a strong resemblance to the arthroscopic tools commonly employed in knee arthroscopic surgeries.^1^ Typically, a 4-mm-diameter, 0° endoscope is utilized in this approach.^1,4^ The surgical interventions are executed through two portals, while maintaining continuous saline irrigation throughout the procedure.^5^

### 2:54 V Point.

Initially, I perform dissection to expose the V point utilizing radiofrequency probes. The V point encompasses the C6 and C7 laminae.

### 3:15 Bone Work.

Following the identification of the V point at C6–7 on the right side, bone manipulation commences at this location. Using a diamond drill and smaller Kerrison rongeurs, a minor ipsilateral laminotomy is conducted on the upper and lower laminae. Additionally, a medial facetectomy is performed to enhance visibility and exposure of the cervical nerve root.

### 3:43 Removal of Ligamentum Flavum.

After ipsilateral lamino-foraminotomy, ligamentum flavum is partially removed to expose the C7 nerve root.

### 3:54 Exposure of Cervical Nerve Root.

I remove cervical epidural membrane and fibro-areolar tissue around a cervical nerve root. The C7 nerve root is now completely exposed.

### 4:12 Axillar Area Dissection.

Complete exposure of the axillary area and nerve root is necessary to effectively remove the ruptured disc particles. Typically, the cervical nerve root comprises both sensory and motor nerve roots.

### 4:31 Removal of Ruptured Disc Fragments.

I extracted a total of six ruptured disc fragments without excessively retracting the dura or nerve root. The initial removal addressed the first ruptured particle, followed by the extraction of an additional five ruptured disc fragments after a thorough dissection of the axillary area. At the level of C6–7 or C7–T1, the cervical nerve roots have a superior origin compared to the disc level.^6^ Moreover, the nerve root emerges in very close proximity to the superior pedicle. When performing cervical foraminotomy in this region, adequate exposure of the axillary segment of the nerve root and disc space is achieved. This allows for complete removal of ruptured disc fragments from the axillary area surrounding the nerve root.

### 5:36 Epidural Drainage Catheter.

Following the sufficient removal of the disc, the surgery concluded with a meticulous examination for any residual disc fragments, conducted using a hook and probe. As a preventive measure against postoperative epidural hematoma, I inserted an epidural drainage catheter. Typically, a single-level posterior cervical foraminotomy procedure of this nature lasts less than 60 minutes. Bleeding is minimal, seldom surpassing 100 ml.

### 6:06 Postoperative MRI.

The postoperative MRI confirmed the comprehensive extraction of ruptured disc particles located at the right C6–7 level. The accomplishment of posterior cervical foraminotomy with discectomy was attained through the utilization of two small skin incisions.

### 6:26 Postoperative Course and Conclusion.

Following the procedure, there was a notable improvement in the patient’s radicular pain. The surgical approach of biportal endoscopic posterior cervical foraminotomy bears resemblance to microscopic surgery, leveraging familiar anatomical principles. This approach presents as a potentially effective minimally invasive spine surgery for alleviating unilateral radicular pain arising from cervical foraminal disc herniation. Considering its efficacy, biportal endoscopic posterior cervical foraminotomy stands as a viable alternative among treatment options for cervical foraminal lesions.

### 7:08 Discussion.

The use of smaller skin incisions compared to microsurgery, along with the employment of slender two portals of smaller diameter than a tubular retractor, potentially minimizes muscle and ligament damage.^1,3^ This approach can lead to reduced postoperative pain and a swifter recovery period.^7^ Additionally, this surgical method has shown promise in reducing intraoperative bleeding. The high-definition and magnified view provided by biportal endoscopy accurately delineates the surgical area, greatly aiding in precise surgical maneuvers. However, it’s worth noting that mastering the surgical techniques in biportal endoscopy typically entails a longer learning curve compared to microsurgery. Nevertheless, individuals who have acquired ample experience in endoscopic lumbar surgery and cervical microsurgery can effectively transition to performing cervical biportal endoscopic surgeries.

## Disclosures

The author reports no conflict of interest concerning the materials or methods used in this study or the findings specified in this publication.

## Supplemental Information

### Patient Informed Consent

The necessary patient informed consent was obtained in this study.
